# Flow cytometric analysis of myelodysplasia: Pre‐analytical and technical issues—Recommendations from the European LeukemiaNet

**DOI:** 10.1002/cyto.b.22046

**Published:** 2021-12-11

**Authors:** Vincent H. J. van der Velden, Frank Preijers, Ulrika Johansson, Theresia M. Westers, Alan Dunlop, Anna Porwit, Marie C. Béné, Peter Valent, Jeroen te Marvelde, Orianne Wagner‐Ballon, Uta Oelschlaegel, Leonie Saft, Sharham Kordasti, Robin Ireland, Eline Cremers, Canan Alhan, Carolien Duetz, Willemijn Hobo, Nicolas Chapuis, Michaela Fontenay, Peter Bettelheim, Lisa Eidenshink‐Brodersen, Patricia Font, Michael R. Loken, Sergio Matarraz, Kiyoyuki Ogata, Alberto Orfao, Katherina Psarra, Dolores Subirá, Denise A. Wells, Matteo G. Della Porta, Kate Burbury, Frauke Bellos, Elisabeth Weiß, Wolfgang Kern, Arjan van de Loosdrecht

**Affiliations:** ^1^ Laboratory Medical Immunology, Department of Immunology Erasmus MC, University Medical Center Rotterdam Rotterdam The Netherlands; ^2^ Department of Laboratory Medicine – Laboratory for Hematology Radboudumc, Nijmegen The Netherlands; ^3^ Laboratory Medicine SI‐HMDS, University Hospitals Bristol and Weston NHS Foundation Trust Bristol UK; ^4^ Department of Hematology Amsterdam UMC, location VU University Medical Center, Cancer Center Amsterdam Amsterdam The Netherlands; ^5^ Department of Haemato‐Oncology Royal Marsden Hospital Sutton Surrey UK; ^6^ Department of Clinical Sciences, Division of Oncology And Pathology, Faculty of Medicine Lund University Lund Sweden; ^7^ Hematology Biology Nantes University Hospital and CRCINA Nantes France; ^8^ Department of Internal Medicine I, Division of Hematology & Hemostaseology Medical University of Vienna Vienna Austria; ^9^ Ludwig Boltzmann Institute for Hematology and Oncology Medical University of Vienna Vienna Austria; ^10^ Department of Hematology and Immunology; and Université Paris‐Est Créteil Assistance Publique‐Hôpitaux de Paris, University Hospital Henri Mondor Inserm U955, Créteil France; ^11^ Department of Internal Medicine University Hospital Carl‐Gustav‐Carus Dresden TU Germany; ^12^ Department of Clinical Pathology and Oncology Karolinska University Hospital and Institute Solna Stockholm Sweden; ^13^ Comprehensive Cancer Centre King's College London and Hematology Department, Guy's Hospital London UK; ^14^ Department of Internal Medicine, Division of Hematology Maastricht University Medical Center AZ, Maastricht The Netherlands; ^15^ Assistance Publique‐Hôpitaux de Paris. Centre‐Université de Paris Cochin Hospital, Laboratory of Hematology and Université de Paris, Institut Cochin, INSERM U1016, CNRS UMR8104 Paris France; ^16^ Department of Internal Medicine Ordensklinikum Linz Barmherzige Schwestern – Elisabethinen Linz Austria; ^17^ Hematologics Inc Seattle WA USA; ^18^ Department of Hematology Hospital General Universitario Gregorio Marañon‐IiSGM Madrid Spain; ^19^ Cancer Research Center (IBMCC, USAL‐CSIC), Department of Medicine and Cytometry Service University of Salamanca, Institute for Biomedical Research of Salamanca (IBSAL) Salamanca Spain; ^20^ Biomedical Research Networking Centre Consortium of Oncology (CIBERONC) Instituto Carlos III Salamanca Spain; ^21^ Metropolitan Research and Treatment Centre for Blood Disorders (MRTC Japan) Tokyo Japan; ^22^ Immunology Histocompatibility Department Evangelismos Hospital Athens Greece; ^23^ Flow Cytometry Unit. Department of Hematology Hospital Universitario de Guadalajara Guadalajara Spain; ^24^ IRCCS Humanitas Research Hospital, Rozzano, Milan, Italy & Department of Biomedical Sciences Humanitas University, Pieve Emanuele Milan Italy; ^25^ Department of Haematology Peter MacCallum Cancer Centre, University of Melbourne Melbourne Australia; ^26^ MLL Munich Leukemia Laboratory Munich Germany

**Keywords:** ELN, flow cytometry, MDS, pre‐analytic issues

## Abstract

**Background:**

Flow cytometry (FCM) aids the diagnosis and prognostic stratification of patients with suspected or confirmed myelodysplastic syndrome (MDS). Over the past few years, significant progress has been made in the FCM field concerning technical issues (including software and hardware) and pre‐analytical procedures.

**Methods:**

Recommendations are made based on the data and expert discussions generated from 13 yearly meetings of the European LeukemiaNet international MDS Flow working group.

**Results:**

We report here on the experiences and recommendations concerning (1) the optimal methods of sample processing and handling, (2) antibody panels and fluorochromes, and (3) current hardware technologies.

**Conclusions:**

These recommendations will support and facilitate the appropriate application of FCM assays in the diagnostic workup of MDS patients. Further standardization and harmonization will be required to integrate FCM in MDS diagnostic evaluations in daily practice.

## 1 INTRODUCTION

Flow cytometric immunophenotyping allows the identification, enumeration, and characterization of hematopoietic cells of distinct cell lineages and their differentiation stages in the bone marrow (BM) and peripheral blood (PB). For these reasons, flow cytometry (FCM) is uniquely placed to aid in diagnosing and prognosticating patients with suspected or confirmed myelodysplastic syndrome (MDS). Over the last decade, numerous publications have addressed immunophenotypic abnormalities in MDS patients. However, implementation of FCM into routine diagnostic activities remains limited due to the lack of a universal consensus on sample types, processing, staining, and data analysis, interpretation and reporting. At the European Leukemia Net (ELN) group meeting on MDS diagnostics in 2006 (Valent, et al., 2007b), it was concluded that there was no generally accepted consensus on uniformly used standard protocols and techniques, and that multicenter projects to standardize and harmonize methodologies and reagents were required. To fulfill this need, the International Myelodysplastic Syndromes Flow Cytometry working group of the ELN (ELN iMDS Flow WG) was convened in 2008 and at that time published the first recommendations (Valent et al., 2010; Valent, et al., 2017c). Since 2008, significant progress has been made in FCM with respect to technical issues (including software and hardware) and pre‐analytical procedures. Additional diagnostic standards and criteria for MDS diagnosis have been proposed and include FCM (Valent et al., 2010; Valent, et al., 2017c).

The purpose of this manuscript is to discuss and provide updates on (1) the optimal methods for sample processing and handling; (2) antibody panels and fluorochromes; and (3) current hardware technologies. Recommendations are made based on the data and expert discussions generated from 13 yearly meetings of the ELN iMDS Flow WG between 2008 and 2020.

## 2 SAMPLES

### 2.1 Source of samples

For FCM analysis of patients with cytopenias and possible MDS, BM is the required sample source. The BM sample should be of high quality with representation of precursor cells, and hemodilution should be prevented as much as possible. Therefore, the BM sample for FCM should preferably be the first aspirate pull and should be between 2 and 3 ml. If this is not possible or larger volumes are needed, it is recommended to reposition the aspiration needle or to perform a separate puncture. It is recommended to estimate the potential contamination with PB, for example, the presence of >90% mature (CD10+) neutrophils or high numbers of T‐cells in case of neutropenia in a BM sample are indicative of significant hemodilution (Aldawood et al., 2015; Brooimans et al., 2009; Delgado et al., 2017; Loken et al., 2009; Nombela‐Arrieta & Manz, 2017; Pont et al., 2018).

Analysis of BM samples allows detailed evaluation of the myeloid precursor cells and multiple maturation stages of the three major cell lineages, namely the granulocytic, monocytic, and erythroid lineage. In addition, less frequent cell types such as B‐cell precursors, basophils, mast cells, eosinophils, and dendritic cells can be analyzed simultaneously. Megakaryocytic cells remain challenging to assess by FCM, due to their large size, low frequency, fragility, and nonspecific binding of platelets to other cell types. Consequently, their analysis is not yet recommended in the routine diagnostic workup of MDS. For the latter lineage evaluation of PB platelets may be feasible (Sandes et al., 2012), but we do not recommend this analysis yet since limited data are available.

Although BM is the preferred sample source, several studies indicate that PB samples may also provide clinically useful information in suspected MDS patients. The antigens CD10, CD11b, CD13, and CD16 may be abnormally expressed on PB neutrophils and may discriminate MDS patients from non‐MDS patients with sensitivities between 73%–93% and specificities of 90%–100% (Aires et al., 2018; Cherian et al., 2005a; Cherian et al., 2005b; Rashidi et al., 2012). While these findings are promising, they have not been widely adopted and are not part of our current recommendations.

### 2.2 Anticoagulant

It is recommended that samples for FCM MDS analysis are aspirated into tubes with heparin as an anticoagulant. Samples may also be collected in EDTA tubes since there will generally be no significant differences between these two anticoagulants. However, EDTA may influence the expression of specific antigens such as CD10, CD11b, CD16, and CD64, especially when samples are not processed immediately (Elghetany & Davis, 2005; Karai et al., 2018; Stachurski et al., 2008).

## 3 PROCESSING OF SAMPLES

### 3.1 Processing time

Ideally, samples should be processed within 24 h. If this is not possible, for example, in a setting of centralized analysis for a multicenter trial, samples should be stored and/or shipped at ambient conditions; storage/shipment at 4°C should be avoided (Alhan et al., 2016). It is well recognized that sample processing within 36 h or even up to 72 h, will be acceptable. Samples processed at later time points may still be evaluable, but extra controls should be included to evaluate the quality of the sample (e.g., check scatter characteristics, percentage of dead cells, expression patterns on normal cells). Each center performing FCM analysis should evaluate analyte stability as part of the validation process (confirm sample integrity and antigen stability).

### 3.2 Lysis versus no‐lysis

#### 3.2.1 Analysis of white blood cells

FCM analysis in patients with MDS currently focuses on white blood cells (WBC) and nucleated red blood cells (NRBC). Due to the high number of mature erythrocytes present in BM samples, the WBCs are usually analyzed after RBC lysis. Lysis of the mature RBC population before antibody staining of the WBC is recommended for two main reasons. Firstly, bulk‐lysis permits the assessment of identical cell suspensions in the separate aliquots of different analysis tubes, thereby facilitating the comparison between tubes. Secondly, this approach allows the use of a fixed cell concentration for antibody incubation. Bulk‐lysis has not been shown to affect the fluorescent signals of the antibodies, if well titrated (Kalina, et al., 2012), does not result in selective loss of cell populations, and does not result in increased levels of debris and/or doublets (Flores‐Montero et al., 2017; Theunissen, et al., 2017). Bulk‐lysis is especially recommended in childhood MDS, in which about 50% of patients have refractory cytopenia of childhood (RCC), frequently with hypocellular BM (Aalbers et al., 2013; Aalbers et al., 2015). For lysis of the nonnucleated RBCs, ammonium chloride (either homemade or commercially available) can be used. Other (commercial) lysing solutions may be used after parallel testing and comparison. Within the EuroFlow protocol, erythrocyte lysis is performed after the antibody incubation phase. This results in the lowest cell loss, and the presence of a fixative prevents the decrease in MFI during the period between staining and data acquisition (Kalina, et al., 2012).

Alternatively, it is possible to adopt a Lysis‐no‐Wash method. With such procedures, aliquots of 50 to 100 μl of whole BM or PB are incubated with the antibody cocktail of each tube. After incubation at room temperature or at 4°C in the dark (because of the light sensitivity of fluorochromes), mature RBCs are lysed and the sample is immediately processed for acquisition by the flow cytometer, without any washing in between (Mathis et al., 2013).

#### 3.2.2 Analysis of immature red blood cells

**FIGURE 1:**
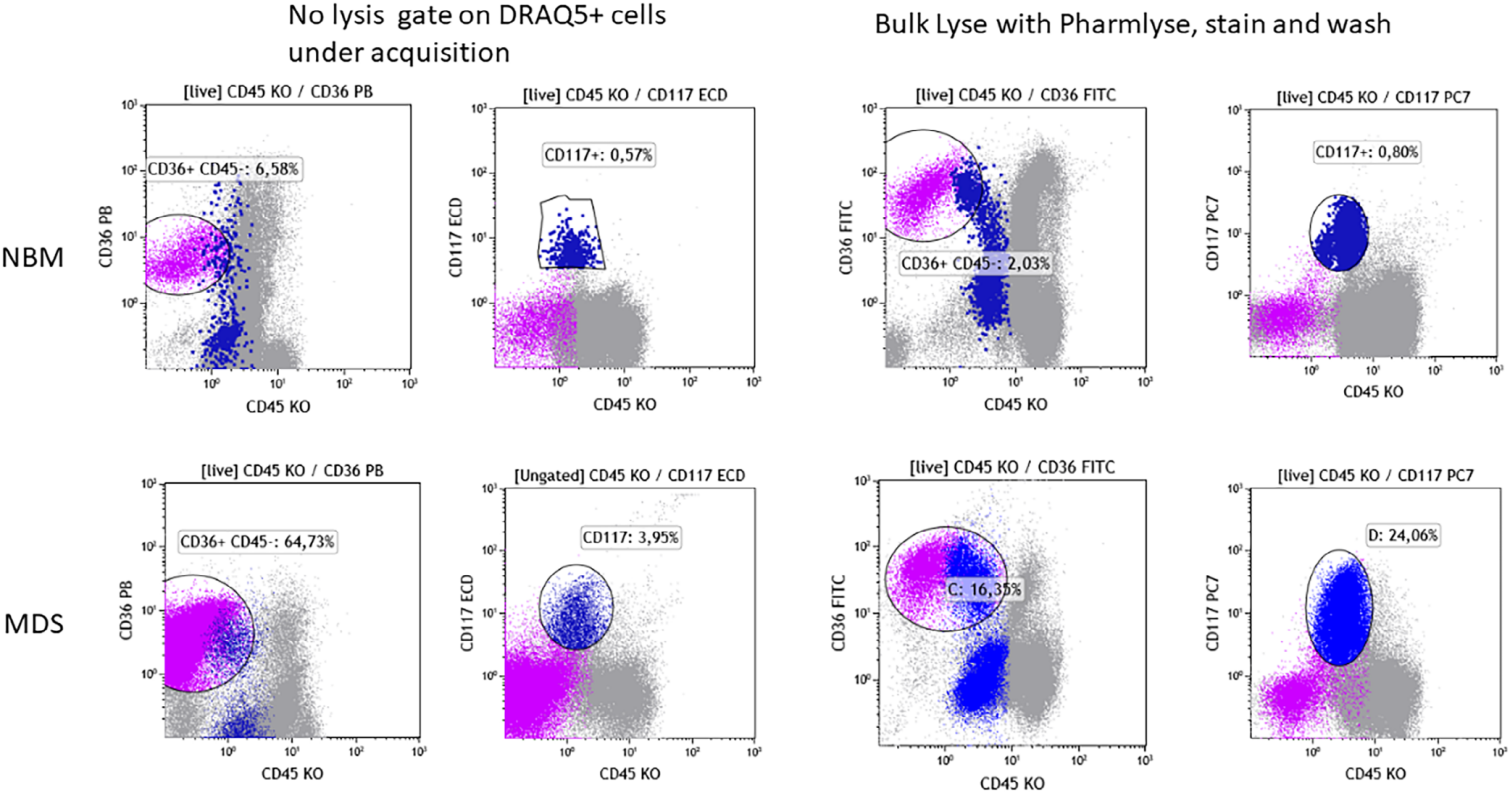
Impact of lysis on the erythroid compartment (CD36 + CD45−, violet dots) and on the frequency of immature myeloid cells (CD117+, dark blue dots). Upper row: Left two plots to show a normal BM sample processed without lysis. Cells were stained with a panel containing CD36 PB, CD45KO, and CD117 ECD. DRAQ5 was added after the staining and the acquisition gate was set on DRAQ5 positive cells. Right two plots: A part of the sample was subjected to bulk lysis (with NH4Cl‐based lysis buffer) and stained with a panel containing CD36FITC, CD45KO, and CD117PC7. Lower row: A BM sample from an MDS patient processed in a similar way [Color figure can be viewed at wileyonlinelibrary.com]

FCM analysis of MDS patients involves not only the granulocytic and monocytic lineages but also analysis of the erythroid cell compartment, which provides additional valuable information (Cremers, et al., 2017; Mathis et al., 2013; Westers, et al., 2017). However, because of the RBC lysis and centrifugation steps, some erythropoietic precursors (EP) may be destroyed as well. Consequently, the percentages of EP by FCM may differ from those obtained by cytological assessment. Previous publications have reported that lysis also alters the light scatter characteristics of EP resulting in smaller, more homogeneous cells compared with erythroid cells obtained by density sedimentation using Ficoll (Machherndl‐Spandl et al., 2013; Wangen et al., 2014). EP morphology was also found to be affected by lysis (Mathis et al., 2013). Of note, the proportion of erythropoiesis detected in non‐lysed BM by routine FCM panels showed better agreement with cytomorphology than data obtained from lysed BM samples (Violidaki et al., 2020). The proportion of myeloid blasts (measured by the frequencies of the CD34+ and CD117+ compartment) can thus be falsely elevated in the lysed BM due in part to the loss of the EP (Figure 1). Most of the abnormalities detected in the erythroid compartment of MDS samples are seen in both lysed and non‐lysed BM and therefore both approaches can be applied. Nevertheless, direct comparison in the same BM sample stained with the same antibody combinations with and without lysis had demonstrated significantly lower MFI and CV values of CD71, CD36, and CD117 in EP when lysis was performed (Violidaki, et al.). Therefore, using non‐lysed whole samples (optionally diluted in phosphate‐buffered saline [PBS]) with an adequate gating strategy thus seems a reasonable and feasible option for an accurate assessment of the erythroid compartment in suspected MDS. Of note, it is recommended to assign one fluorescence channel to a nuclear dye (e.g., DRAQ5), allowing the exclusion of nonnucleated elements, that is mature erythrocytes, by gating during acquisition.

#### 3.2.3 Impact of dead cells

Due to the pre‐analytical processing and depending on the type of lysis used, the viability of some cell subsets may be affected, resulting in an increase of the dead‐cell fraction (debris). The bulk‐lysis protocol developed by EuroFlow did not result in increased percentages of debris or doublets, although cell viability was not explicitly investigated (Flores‐Montero et al., 2017; Theunissen, et al., 2017). Some dead cells may nonspecifically bind monoclonal antibodies and/or the fluorochrome conjugate, resulting in higher background staining. The optimal way to assess only viable nucleated cells is to use a nuclear viability dye, such as 7AAD or DRAQ7 (Shenkin et al., 2007). This also allows exclusion of erythroid cells that have lost their nucleus. Alternatively, amine‐staining dyes have recently been investigated; they bind cell membrane‐bound amines that are highly increased on dead cells. The amine‐staining dyes thereby allow only to examine the live cell population for analysis (Perfetto et al., 2006). At this moment, we do not recommend the addition of viability dyes for the analysis of leukocytes, but this issue will be evaluated and discussed within future ELN iMDS Flow WG meetings.

## 4 PANEL

**FIGURE 2:**
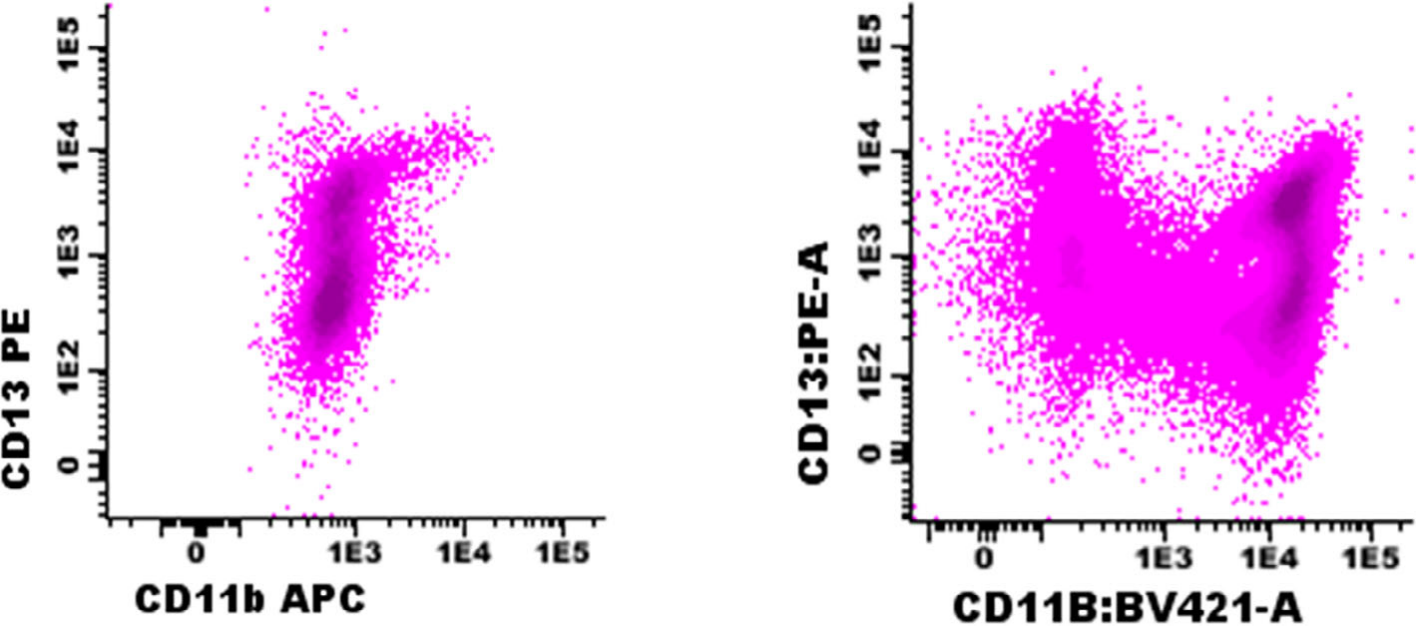
Example of CD13 versus CD11b demonstrating granulocytic maturation in a BM aspirate tested with suboptimal antibody combinations (left panel; CD11b clone D12, APC) and a validated panel (right panel; CD11b clone 10.1, BV421). Cells were gated based on CD45‐SSC and resulting granulocytic cells are shown in the plots. In the right plot, variable expression of CD11b can be observed, with the highest CD11b expression on the most mature granulocytes. In contrast, in the left plot, the staining is too weak and does not allow separation of CD11b‐negative and CD11b‐positive cells. Since APC is a bright fluorochrome, this weak staining more likely is related to the applied clone or to an inappropriate antibody titer [Color figure can be viewed at wileyonlinelibrary.com]

Various groups have published recommendations on panel design and have been proscriptive in stipulating antigen and fluorochrome combinations (Lacombe et al., 2016; van Dongen, et al., 2012). While our guidelines have not been devised that way, it is critical that when individuals or groups of laboratories implement panels, extensive validation, verification, and optimization steps are required to ensure that antigenic patterns are consistent in normal BM samples and appropriate cytopenic controls. Confirmation of staining using positive and negative controls is insufficient as the intensity and maturation patterns are critical (Figure 2). Unless there is an unavoidable reason for locally developed panels to be tested, we recommend a fully validated and published combination be used.

Antibodies that are informative of dysplastic features are summarized in Table 1, and their diagnostic value is explained in detail in a separate paper in this issue of Clinical Cytometry. These antibodies are combined in FCM panels that produce a maximum of information about differentiation of specific subsets for example CD34/CD117/HLA‐DR, CD13/CD11b/CD16, and CD105/CD71/CD36 for myeloid progenitor, neutrophilic and erythroid maturation, respectively. CD45 should be included as a minimum backbone in every tube. Increasing the number of recurring (backbone) markers in every tube facilitates more accurate gating procedures upon analysis of the cellular compartments of the BM (van Dongen, et al., 2012). The exact composition of the antibody panel may depend on local guidelines, available equipment, core facilities, and resources. Therefore, we provide general FCM guidelines but do not propose strict recommendations for specific antibodies and fluorochromes. Nevertheless, we recommend using at least eight‐color panels to allow optimal gating strategies and evaluation of all relevant cell populations. Various fluorochrome‐antibody conjugates can be used, but the general principles for panel design and antibody titration should be taken into account. Examples of two well‐validated and commonly used panels for evaluation of MDS patients are shown in Tables 2 and 3 (Porwit & Rajab, 2015; van Dongen, et al., 2012). All antibodies used should undergo quality control and internal validation testing before its first use, to ensure comparable fluorescence intensities between antibody lots and over time.

**TABLE 1:** Recommended antibodies for FCM analysis of various cell types

Cell subset	Backbone markers	Recommended markers	Optional
Myeloid progenitor	CD45, CD34, CD117, HLA‐DR	CD13, CD33, CD10, CD11b, CD15, CD38, CD7, CD56	TdT, CD5, CD19, CD25, CD133
Lymphoid progenitor	CD45, CD34	HLA‐DR, CD10, CD19	CD22
Granulocyte	CD45, CD117	HLA‐DR, CD13, CD33, CD11b, CD16, CD10, CD15, CD14, CD64, CD56	CD34, CD5, CD7
Monocyte	CD45	HLA‐DR, CD13, CD33, CD11b, CD14, CD34, CD36, CD64, CD16, CD56, CD117	CD2, MDC8 (Slan), CD300e
Erythroid	CD45, CD34, CD117	HLA‐DR, CD36, CD71, CD105, CD13, CD33	CD235a
**Optional cell subsets for analysis:**			
Basophil	CD45	CD123, HLA‐DR	CD203c
Mast cell	CD117	CD45, HLA‐DR	CD2, CD25
Dendritic cell	CD45, CD34, CD117	HLA‐DR, CD123	CD11c CD1c, CD141, CD303

**TABLE 2:** Example of eight‐color FCM panel for analysis of MDS patients: EuroFlow (van Dongen, et al., 2012)

	PB	PO	FITC	PE	PerCP Cy5.5	PC7	APC	APC H7	Aim
1	HLADR	CD45	CD16	CD13	CD34	CD117	CD11b	CD10	Focused on granulocytic lineage
2	HLADR	CD45	CD35	CD64	CD34	CD117	IREM2	CD14	Focused on monocytic lineage
3	HLADR	CD45	CD36	CD105	CD34	CD117	CD33	CD71	Focused on erythroid lineage
4	HLADR	CD45	TdT	CD56	CD34	CD117	CD7	CD19	Aberrant expression of lymphoid markers

Abbreviations: APC, allophycocyanin; APC‐H7, allophycocyanin‐Hilite; FITC, fluorescein isothiocyanate; PB, Pacific Blue; PC7, phycoerythrin cyanin 7; PE, phycoerythrin; PerCP‐Cy5.5, phycoerythrin cyanin 5.5; PO, Pacific Orange.

**TABLE 3:** Example of 10‐color FCM panel for analysis of MDS patients: Canada (Porwit & Rajab, 2015)

	FITC	PE	ECD	PC5.5	PC7	APC	APC‐F700	APC‐F750	PB	KO
1^a^	CD4/Kappa	CD8/Lambda	CD3/CD14	CD33	CD20/CD56	CD34	CD19	CD10	CD5	CD45	
2	CD65	CD13	CD14	CD33	CD34	CD117	CD7	CD11b	CD16	CD45	
3	CD36	CD64	CD56	CD33	CD34	CD123	CD19	CD38	HLA‐DR	CD45	
4	CD71	CD11c	CD4	CD33	CD34	CD2	CD10	CD235a	CD15	CD45	

*Note*: Tube 1 is meant for screening, tube 2–4 for more detailed MDS analyses.

Abbreviations: APC, allophycocyanin; ECD, phycoerythrin‐Texas red‐X; FITC, fluorescein isothiocyanate; KO, Krome Orange; PB, Pacific Blue; PC5.5, phycoerythrin cyanin 5.5; PC7, phycoerythrin cyanin 7; PE, phycoerythrin.

^a^ Screening tube.

Notably, the markers recommended only identify those that have been validated in a multicenter setting. Several studies have introduced markers that may be of additive value, either as a single marker, in combination with a diagnostic mini‐panel or in combination with other markers (Alayed et al., 2020; Della Porta et al., 2012; Mestrum et al., 2021; Shameli et al., 2020).

## 5 FIXATION OF SAMPLES

For samples processed with the stain‐lyse‐wash method, we recommended to fix the cells after staining and washing in order to stabilize cell membranes, prevent possible dissociation of antibodies and reduce biohazard (Lanier & Warner, 1981). Washed cells should be suspended in fixation buffer (0.5% paraformaldehyde [PFA] in PBS [pH 7.4] is recommended) and preferably be acquired within 1 h. If unavoidable, they can be kept at 4°C in the dark, up to 24 h.

## 6 FLOW CYTOMETER INSTRUMENT SET‐UP

MDS assays require appropriate instrument settings, similar to other FCM assays for hematological malignancies. Each cytometer should be set up and calibrated according to the supplier's recommendation, generally including setup beads. It is recommended to set up the instrument further using either the EuroFlow approach or the Harmonemia approach (Kalina, et al., 2012; Lacombe et al., 2016). Both approaches use specific fluorescence targets to generate highly comparable data between different cytometers and between different laboratories. Some current cytometers allow the exchange of assays including information about instrument set‐up, allowing the cytometer to automatically set itself up so that highly comparable data can be obtained. A strict daily quality assessment should be performed according to the supplier's recommendation.

The stability of the cytometer's performance over time is essential for MDS analysis. This is because many parameters in the FCM MDS score depend on measuring relatively discrete changes that rely on preset reference ranges and ratios. For example, overexpression of CD117 on myeloid progenitors may be defined based on the populations' CD117 median fluorescent intensity crossing a preset threshold. Thresholds and ratios are based on previous analyses of normal, reactive, and pathological BM control samples. Hence, stable longitudinal performance is essential, and when changes occur in cytometer output for any reason, re‐verification of the preset reference ranges is required. Compensation settings can potentially be challenging due to the simultaneous analysis of several cell subsets with different autofluorescence and compensation requirements. While compensation may require updating at regular intervals, the analysis strategy used should be locked down.

## 7 DATA ACQUISITION

After processing and staining the sample, the cells should be acquired as soon as possible, preferably within 1 h of completing the staining procedure (Alhan et al., 2016; Kalina, et al., 2012). If cells cannot be measured immediately, they should be stored at 4°C in darkness (see the section on fixation).

In addition to fluorescence channels, forward scatter (FSC) and side scatter (SSC), it is recommended to include FSC‐Height to exclude cell doublets and to include the time parameter to check and ensure the stability of sample acquisition/flow.

In order to have reliable analysis of rare cell populations, a minimum of 100,000 WBCs should be acquired per tube with a minimum of 250 CD34+ cells. For samples processed according to lyse‐no‐wash methods (e.g., for evaluation of monocytes in PB) or stain‐no wash methods (e.g., evaluation of erythroid lineage) appropriate gates should be set and sufficient cells should be acquired in order to perform reliable analysis (e.g., 10,000 monocytes or 100,000 erythroid cells).

**FIGURE 3:**
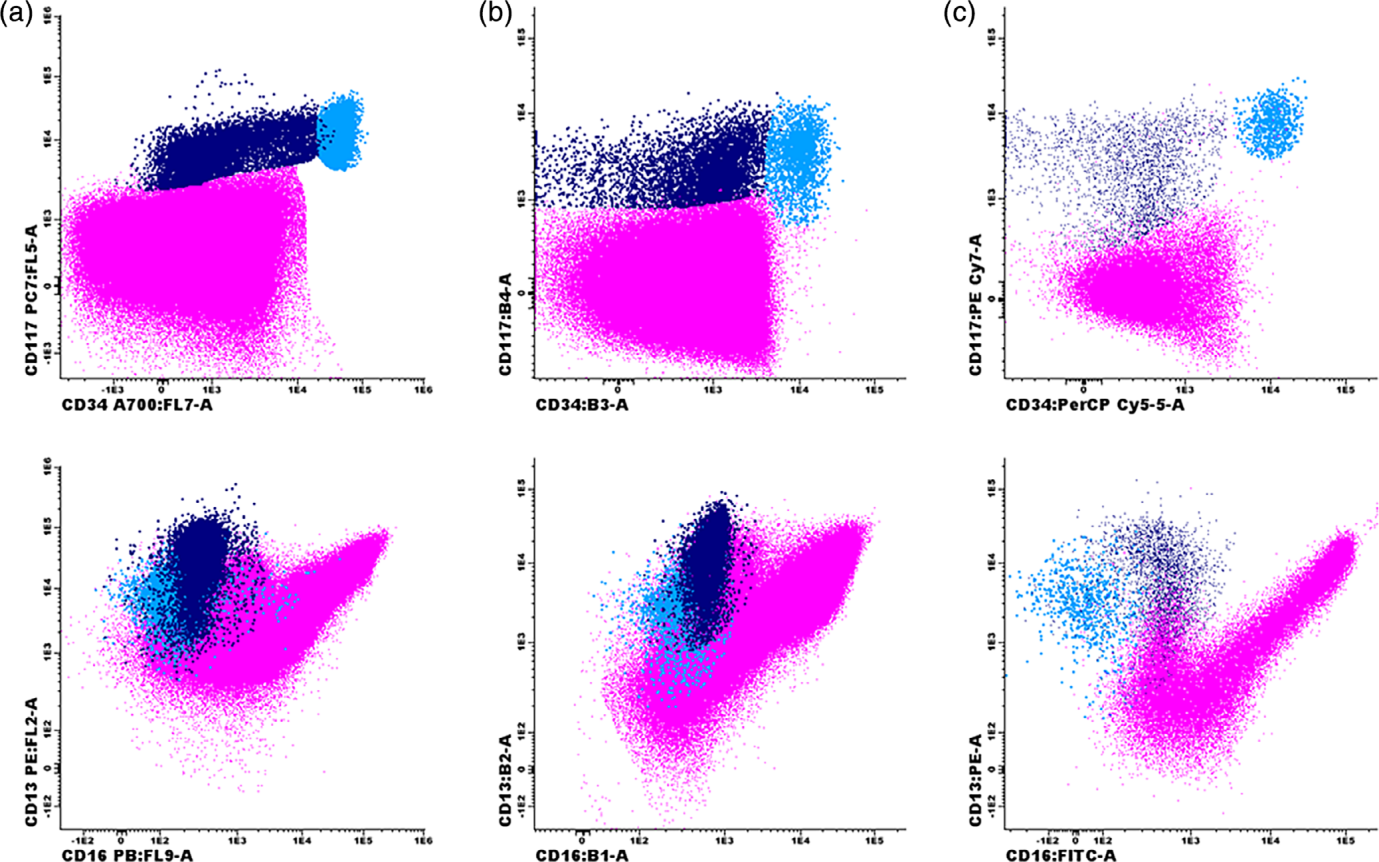
Example of granulocytic maturation in normal bone marrow samples stained and processed according to different protocols. (a) Lyse‐wash‐stain‐wash‐fix‐acquire on Navios protocol, labeling CD56‐FITC/CD13‐PE/CD14‐ECD/CD10‐PC5.5/CD117‐PC7/CD11b‐APC/CD34‐A700/CD33‐A750/CD16‐PB/CD45‐KO. (b) Lyse‐wash‐stain‐wash‐acquire on FACS Canto protocol, labeling CD45‐V500c/HLADR‐BV421/CD16‐FITC/CD13‐PE/CD34‐PerCP‐Cy5.5/CD117‐PC7/CD33‐APC/CD11b‐APCCy7. (c) Stain‐lyse‐wash‐acquire on FACS Canto protocol, labeling CD45‐PO/HLADR‐PB/CD16‐FITC/CD13‐PE/ CD34‐PerCP‐Cy5.5//CD117‐PC7/CD11b‐APC/CD10‐APCH7. CD34+/CD117+ myeloid progenitors are shown in light blue; promyelocytes (CD34‐/CD117+/CD13+) are shown in dark blue; granulocytes are shown in purple [Color figure can be viewed at wileyonlinelibrary.com]

Obviously, the final data are dependent on sample processing, antibody panel, and instrument settings. Figure 3 shows an example of FCM data of granulocytes and their precursors in normal BM samples obtained in three different laboratories, showing similarities and differences in the FCM results. As indicated before, it is most critical that the same assay is being applied within a laboratory and that this assay has well been validated using appropriate controls.

## 8 PRE‐ANALYTICAL ISSUES RELATED TO SPECIFIC CELL POPULATIONS

In addition to the general pre‐analytical recommendations and considerations reported above, there are some explicit technical and pre‐analytical recommendations for specific cell populations.

### 8.1 Granulocytes

The most commonly encountered problem encountered when analyzing maturing myeloid cells is hemodilution. This results in a right shift, towards the more mature neutrophilic cells (CD16++/CD11b++/CD13++/CD15++). Furthermore, a processing time longer than 24 h can account for changes of antigen expression, for example CD16 or CD11b, and scatter properties of cells due to aged BM specimen (Alhan et al., 2016; Loken et al., 2008; Loken & Wells, 2008). Aged cells present with a clearly lower CD11b and a slightly lower CD16 expression intensity. In addition to affecting marker expression, a delay in the time between staining and acquisition causes an increase in the sideward scatter (SSC) signal of neutrophils, and storage of stained samples at 4°C further enhances this effect (Alhan et al., 2016).

### 8.2 Monocytes

FCM characterization of monocytic maturation and aberrant antigen expression is of particular value as the assessment of promonocytes by cytomorphology can be challenging (possible minor differences between promonocytes and atypical mature monocytes or blast cells). When performing FCM assessment of the monocyte lineage special attention should be paid to autofluorescence as this is more pronounced in this lineage. The most common abnormal antigen expression seen on the monocyte lineage is CD56 (see separate paper on our multicenter study in this issue of Clinical Cytometry). It is critical that each laboratory establishes its own cutoff levels for the increased autofluorescence detected. Furthermore, it is well established that monocytes (also granulocytes and myeloid progenitor cells) can upregulate CD56 following treatment and that CD56 may also be increased during infection. Therefore, caution should be exercised when assessing such immunophenotypic abnormalities, particularly in posttreatment samples.

**FIGURE 4:**
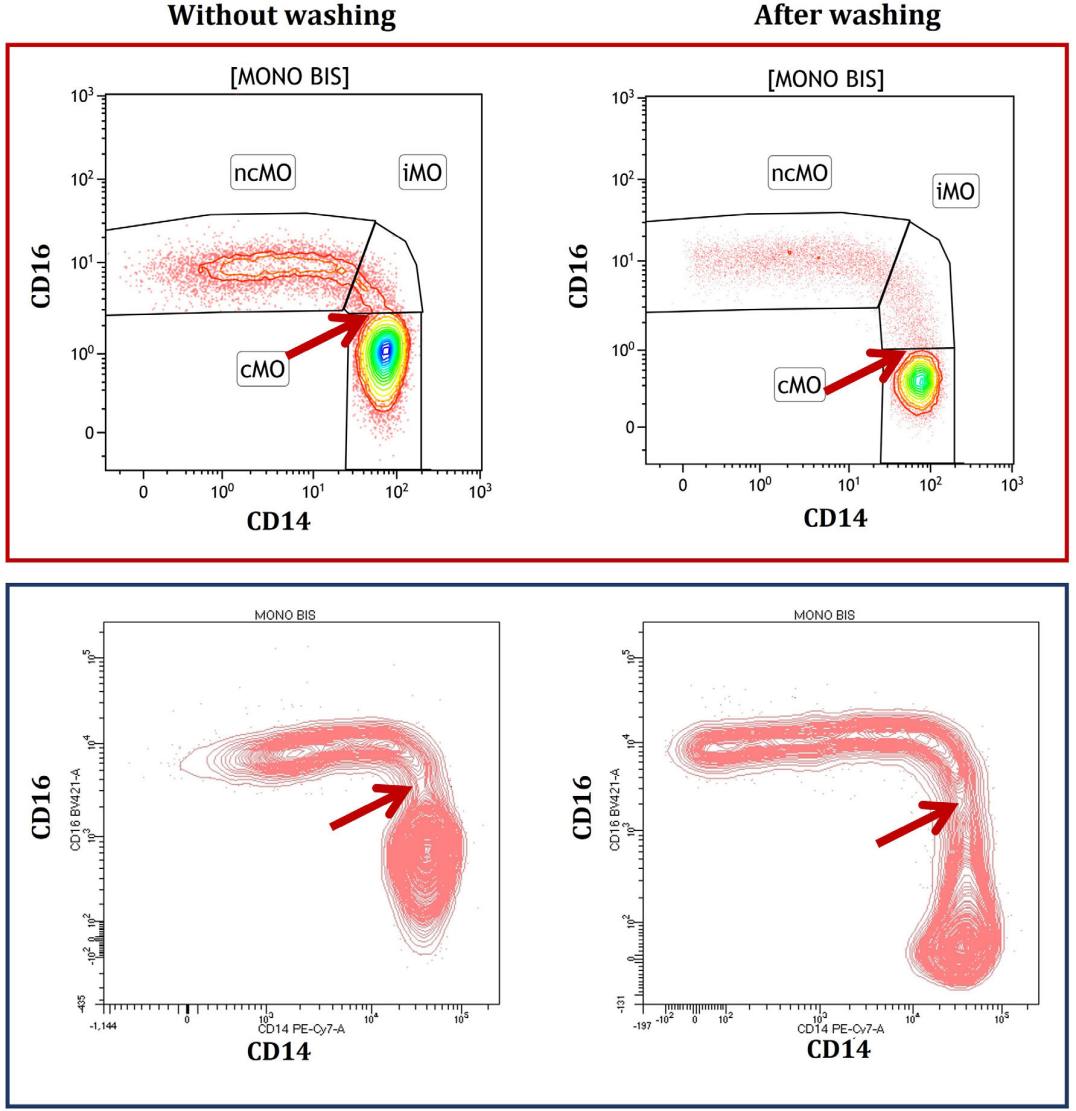
Example of the same sample from a healthy blood donor analyzed without (left panels) or with (right panels) washing procedure on two different flow cytometers from different manufacturers (upper and lower panel) [Color figure can be viewed at wileyonlinelibrary.com]

For the identification of patients with possible CMML, the “monocyte assay” can be used to analyze monocyte subsets based on the expression of CD14 and CD16 (Selimoglu‐Buet, et al., 2015). For this assay, fresh whole blood samples collected in EDTA should be used, with a volume of 200 μl to ensure the acquisition of at least 10,000 events of classical monocytes. Samples should be processed immediately following a lyse‐no‐wash approach, since washing steps may lead to lower CD16 staining, likely due to the weak affinity of the antibody, which may hamper appropriate gating of the classical monocytes population (see Figure 4 for illustrative case).

### 8.3 Erythroid cells

As previously described, sample preparation is important for the analysis of the erythroid lineage. Our multicenter study (Westers, et al., 2017) demonstrated that erythroid parameters obtained from lysed BM samples could contribute to the evaluation of dysplasia in suspected MDS. These markers concerned the coefficient of variance (CV) of the expression of CD36 and CD71 (both increased in MDS), the expression of CD71 (decreased), and the percentage of CD117+ erythroid precursors (decreased or increased). The application of these markers in a separate cohort confirmed their validity in the FCM analysis of MDS (Cremers, et al., 2017). Analysis of non‐lysed BM revealed differences in subset distribution, expression levels, and CV values. Yet, identified markers in the non‐lysed setting remain informative for MDS‐associated dysplasia. Validated reference values must be applied to the sample preparation method used in that respective laboratory.

### 8.4 Rare cell types

In addition to the major cell lineages studied in MDS, rare myeloid cells may be examined by FCM as well, including basophils, eosinophils, and mast cells (Valent, Horny, et al., 2007; Valent, Orazi, Steensma, et al., 2017). These cells are analyzed, depending on clinical, histopathological, cytogenetic, and molecular data (Valent, Horny, et al., 2007; Valent, Sotlar, Blatt, et al., 2017). For example, in patients with a known mast cell disease, elevated tryptase, or an identified *KIT* mutation, mast cells have to be examined in order to exclude or confirm the presence of a concomitant systemic mastocytosis (SM‐MDS) (Valent, Akin, et al., 2007; Valent, Akin, Hartmann, et al., 2017; Valent, Akin, & Metcalfe, 2017). For mast cell analysis, it is of utmost importance that the BM aspirate sample is of optimal quality and that the cells are analyzed within 12 h (Valent, Akin, et al., 2007). Aberrant expression of CD2, CD25, and/or CD30 on mast cells is a minor diagnostic criterion of SM and if present, suggests a diagnosis of SM (Valent, Akin, et al., 2007; Valent, Akin, Hartmann, et al., 2017; Valent, Akin, & Metcalfe, 2017). In patients with suspected concomitant mast cell leukemia (MCL), the same markers can be applied (Valent, Akin, et al., 2007; Valent, Akin, Hartmann, et al., 2017; Valent, Akin, & Metcalfe, 2017; Valent et al., 2014). Differential diagnoses are myelomastocytic leukemia (MML) (Valent et al., 2014) and basophilic leukemia (Valent, Sotlar, Blatt, et al., 2017). Both conditions may be detected in advanced MDS but are usually not seen in low‐risk MDS. In patients with MML, mast cells account for at least 10% of all cells in the BM or PB smear (Valent et al., 2014). However, in contrast to MCL, SM criteria are not fulfilled (e.g. KIT D816V mutation is not detected) and mast cells usually stain negative for CD2, CD25, and CD30 (Valent et al., 2014). Basophilic leukemia may present as acute or chronic secondary basophilic leukemia in MDS (Valent, Sotlar, Blatt, et al., 2017). In contrast to mast cells, basophils usually display high levels of CD203c and low levels of CD117 (Valent, Sotlar, Blatt, et al., 2017). It is also worth noting that basophils are sometimes elevated in patients with high‐risk MDS even if no basophilic leukemia can be detected (basophils account for less than 40%). Basophilia is of prognostic significance in these cases, and the same holds true for eosinophilia (Matsushima et al., 2003; Valent, Sotlar, Blatt, et al., 2017; Wimazal et al., 2010). Therefore, FCM can also be clinically helpful to determine the numbers (percentages) of basophils and/or eosinophils in BM samples, for example, when a dry tap was obtained. Another indication for eosinophil immunophenotyping is suspected eosinophilic leukemia or a myeloid neoplasm with eosinophilia and rearranged PDGFR or FGFR.

## 9 HARDWARE

The number of lasers and detectors, the setup of the filters, and digital or analog data processing will affect the number of antibodies and the types of fluorochromes that can be assessed simultaneously and will significantly impact the fluorescence patterns obtained. Several original and seminal papers pioneering FCM MDS analysis and scoring date back to the period before multi‐laser and multicolor cytometers were used in routine diagnostic laboratories (Ogata et al., 2002; Stetler‐Stevenson et al., 2001; van de Loosdrecht et al., 2008; van Lochem et al., 2004; Wells et al., 2003). Since multicolor FCM has been applied, more detailed identification of antigen expression and maturation patterns on strict antigen‐defined cell subsets has become possible. For example, defining the monocyte population in a BM from immature CD117 + HLA‐DR++ cells to mature CD14 + CD300e + cells and also tracking aberrancies and difference‐from‐normal patterns, probably requires more than six antigens per analysis tube to ensure the purity of the population studied (van Dongen, et al., 2012). The current recommended score (see accompanying analytical paper) is based on data derived from 6 to ≥8 color studies.

Technologies are now available that allow for a much higher number of antigens to be studied simultaneously. This includes mass and spectral cytometry. However, their application in the context of myelodysplasia is still minimal. One study that used mass cytometry was recently published and highlighted potential new useful parameters that need further investigation (Behbehani et al., 2020). Whether the diagnostic or prognostic power of FCM will increase by “deep immunophenotyping” approaches has yet to be demonstrated. The hematopoietic cell immunophenotype is known to differ between clonal and nonclonal cytopenias. An increased number of antigens may improve the diagnostic and prognostic contribution of FCM if robust evaluation studies are carried out. However, no matter how much data are available, its clinical usefulness relies on the ability to translate this research into practicable service delivery. Therefore, multiparameter software solutions for both current and future hardware and panels are required (Costa et al., 2006; Lhermitte et al., 2018; Pedreira, et al., 2008; Pedreira, Costa, Arroyo, et al., 2008; Saeys et al., 2016; Van Gassen et al., 2015; Van Gassen et al., 2016); these will be discussed in more detail in the accompanying manuscript on the analytical issues.

## 10 CONCLUSIONS

FCM can contribute to the diagnosis and prognostication of MDS patients. To obtain meaningful and comparable inter‐laboratory data, pre‐analytical sample handling is critical. We recommend a lyse‐stain‐wash‐fix protocol for analyzing leukocytes; different protocols are still being used for analyzing erythroid cells. Unfortunately, there is still no standardized protocol that is generally adopted and there are no strict minimal criteria for FCM analysis in MDS. This may partly be due to the rapid technological progress in the field (ability to detect an increasing number of parameters simultaneously) and the complexity of quantitative analysis of the FCM data by current software approaches. Novel software tools will further facilitate and improve the analyses of MDS data and will likely contribute to more common and standardized data sets. The pre‐analytical recommendations provided by our network remain the basis for current and future FCM MDS assays and should contribute to improved and implementable laboratory diagnostics for MDS patients. Further standardization and harmonization will be essential to implementing FCM in routine diagnostic evaluations in MDS, a major focus of our ELN iMDS Flow WG.

## AUTHOR CONTRIBUTIONS

All persons listed as coauthors contributed to preconference and post‐conference discussions and actively participated in the Standardization Conferences. All coauthors contributed equally by discussing criteria, standards and recommendations at the Working Conference: 14th/2nd virtual annual ELN MDS Flow Working Conference April 30, 2021; 13th/1st virtual meeting November 6, 2020; 12th ELN MDS Flow Working Conference October 31–November 2, 2019, Nijmegen, Netherlands; 11th Annual meeting November 1–3, 2018, Munich, Germany; 10th Annual meeting, November 2–4, 2017, Lund, Sweden; 9th Annual meeting, October 27–29, 2016, Paris, France; 8th Annual meeting October 29–31, 2015, Athens, Greece. In addition, all persons listed as coauthors provided essential input by drafting parts of the manuscript and approving the document's final version.
